# Arthroscopic Saucerization and Anatomic Repair of a Wrisberg Variant Discoid Lateral Meniscus: A Physeal‐Sparing Knotless Anchor Technique

**DOI:** 10.1002/atn2.70319

**Published:** 2026-10-18

**Authors:** Stephen Rossettie, Ann Hu, Dan Griffin, Greg Perraut, Ryan Murray

**Affiliations:** ^1^ Department of Orthopedic Surgery MedStar Georgetown University Hospital Washington District of Columbia U.S.A.; ^2^ Georgetown University School of Medicine Washington District of Columbia U.S.A.

## Abstract

Discoid lateral meniscus (DLM) is a congenital variant of the knee characterized by an abnormally shaped meniscus prone to tearing and instability. When symptomatic, the treatment plan is based on the location and pattern of the tear, as well as the presence of instability. In most cases, posterior horn instability is present, but some meniscotibial or root attachments remain. This technical note describes a comprehensive arthroscopic approach to a Wrisberg variant DLM, with a complete absence of posterior root and lateral capsular attachments. A comprehensive arthroscopic reconstruction using a physeal‐sparing, knotless anchor technique is described to recreate the posterior root attachment while avoiding danger to the growth plate that can result from standard transtibial drilling. This anchor‐first, retensionable method allowed for precise adjustment of root tension. Meniscal saucerization, all‐inside and inside‐out peripheral stabilization, and biologic augmentation through microfracture were performed to restore hoop stress transmission and long‐term function. This approach highlights a growth plate‐conscious than standard DLM repair in a uniquely unstable DLM variant.

## Graphical abstract

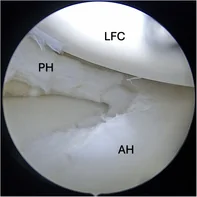

**VIDEO 1:** The video of a stepwise approach to arthroscopic repair of a Wrisberg variant discoid lateral meniscus in the left knee of a pediatric patient in the supine position. Video content can be viewed at https://doi.org/10.1002/atn2.70319.

Discoid lateral meniscus (DLM) is a structural knee variant in which the lateral meniscus is abnormally wide and thick, increasing its susceptibility to tearing and instability. It often presents itself in young pediatric patients.^1^ The Watanabe classification serves to categorize DLMs based on their morphology and attachment patterns, with type 3, the Wrisberg variant, being the most rare and unstable because of the lack of a normal posterior root and meniscal attachments. Instead, the ligament of Wrisberg may be the sole tether, resulting in a hypermobile lateral meniscus.^2^

Standard root repair typically involves a transtibial pullout suture technique.^3^ However, in skeletally immature patients, transphyseal drilling poses a potential risk to the tibial physis and can cause growth arrest or deformities in the future. To preserve the integrity of the physis, physeal‐sparing strategies have been increasingly explored across pediatric knee surgery. One study described a modified percutaneous pullout transverse tunnel technique for tibial spine fractures specifically designed to minimize tibial epiphyseal injury in pediatric patients.^4^ They showed comparable clinical outcomes versus conventional transtibial techniques, supporting the concept that physeal protective approaches may provide equivalent functional outcomes. Although literature specifically evaluating physeal risk in meniscus root repair is limited, data from pediatric anterior cruciate ligament reconstruction provide important context. Prior imaging and simulation studies have shown that transphyseal drilling produces measurable zones of physeal injury, with factors such as tunnel diameter directly influencing the amount of physeal disruption.^5^, ^6^ Techniques that avoid transphyseal drilling may therefore be advantageous in pediatric patients. Accordingly, this report details a physeal‐sparing technique for reconstruction of a completely detached posterior meniscal root in a Wrisberg variant DLM, using a knotless, retensionable anchor.

## SURGICAL TECHNIQUE

The step‐by‐step approach and technique are shown in detail in Video 1.

### Portal Placement and Diagnostic Arthroscopy

The patient is positioned supine with a lateral post. A tourniquet is applied, and the leg is prepped and draped in the usual sterile fashion. Standard anterolateral and anteromedial portals are established for diagnostic arthroscopy. Subsequent diagnostic arthroscopic evaluation reveals a complete DLM with the absence of lateral and posterior capsular attachments and posterior root attachment. The meniscus exhibits global instability and abnormal mobility, particularly posteriorly. The patellofemoral cartilage, medial compartment, meniscal roots, intercondylar notch, and articular cartilage are inspected and confirmed to be intact.

### Meniscus Saucerization

Using an arthroscopic shaver and a basket punch, the central portion of the meniscus is saucerized to convert the discoid into a more normal‐appearing morphology (Figure 1).

**FIGURE 1:**
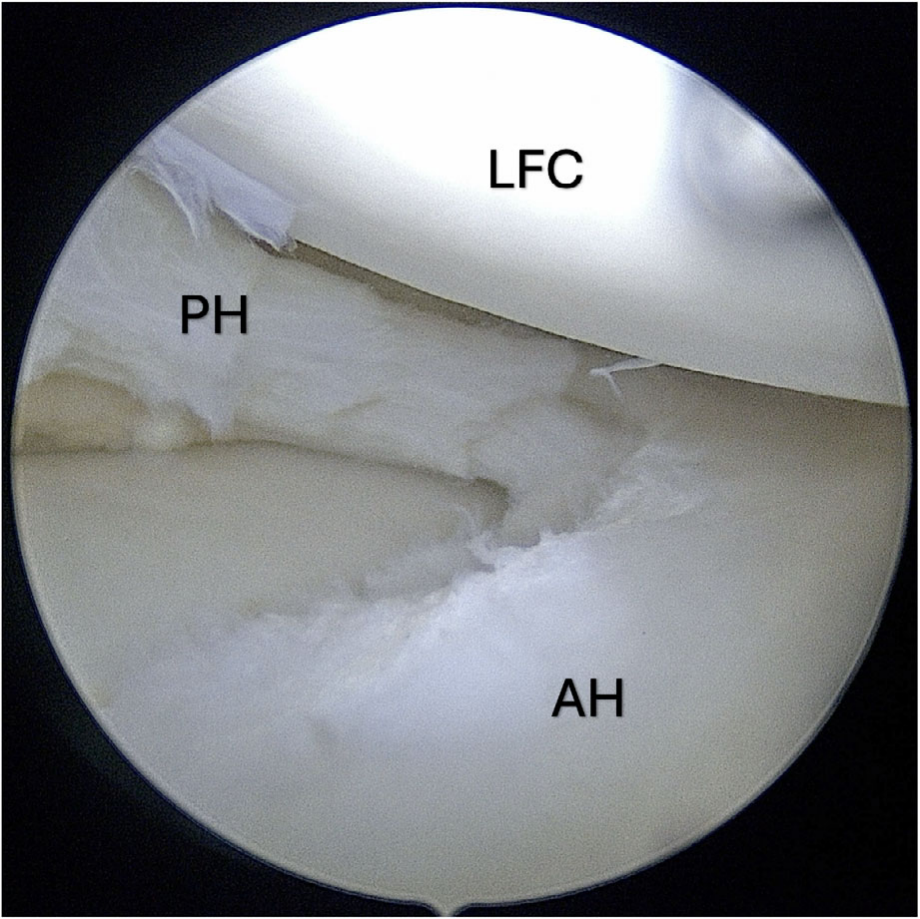
Left knee viewed from a supine, standard anterolateral portal showing a discoid lateral meniscus with the AH, PH, and LFC labeled. Using an arthroscopic shaver and basket punch, the central portion of the meniscus is saucerized to convert the discoid morphology into a more normal semilunar configuration. (AH, anterior horn; LFC, lateral femoral condyle; PH, posterior horn.)

### Posterior Root Reconstruction

The native posterior root insertion site on the lateral tibial plateau is debrided using an arthroscopic rasp and shaver. A high anteromedial portal is created to facilitate instrumentation and to allow for a better trajectory to the native posterior root footprint on the tibial plateau. A curved guide is introduced through the high anteromedial portal. A 1.8‐mm knotless FiberTak soft anchor (Arthrex, Naples, FL) is inserted. The repair suture is passed through the meniscus posterior horn in a horizontal mattress fashion and secured into the knotless anchor, thereby recreating the root attachment (Figures 2, 3, 4).

**FIGURE 2:**
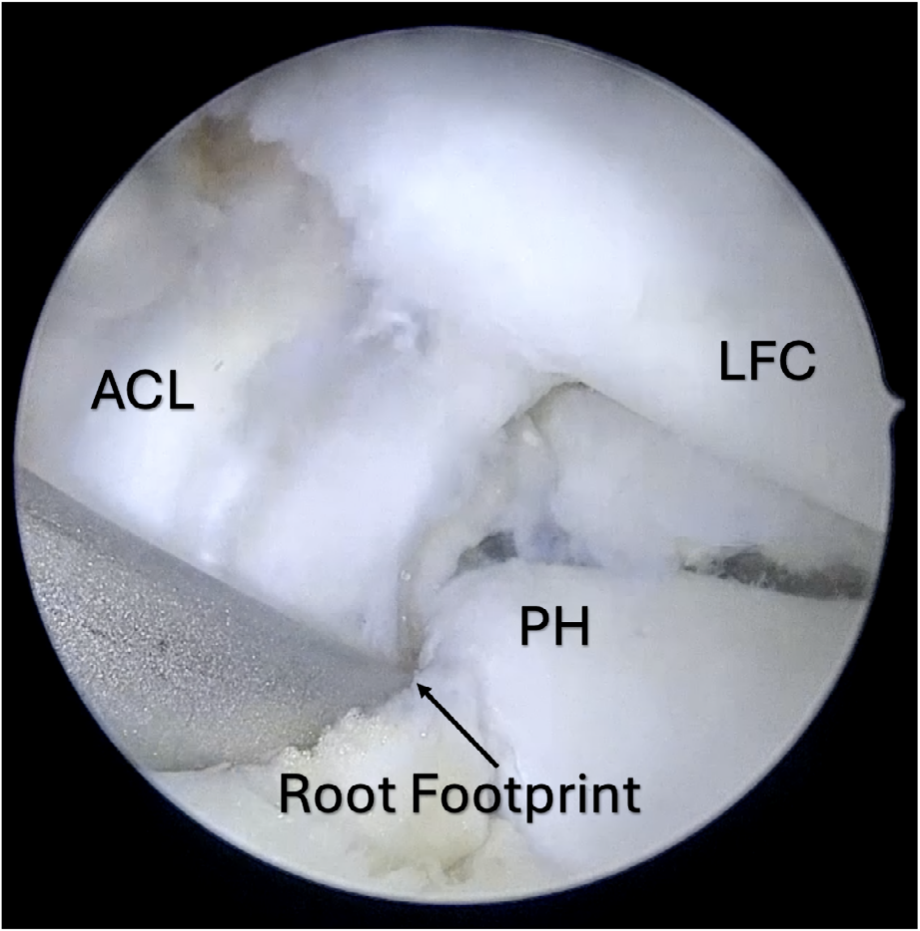
Left knee viewed from a supine, standard anterolateral portal showing the arthroscopic shaver (entered from the high anteromedial portal) debriding the posterior root footprint prior to anchor placement, with the PH, ACL, and LFC labeled for orientation. (ACL, anterior cruciate ligament; LFC, lateral femoral condyle; PH, posterior horn.)

**FIGURE 3:**
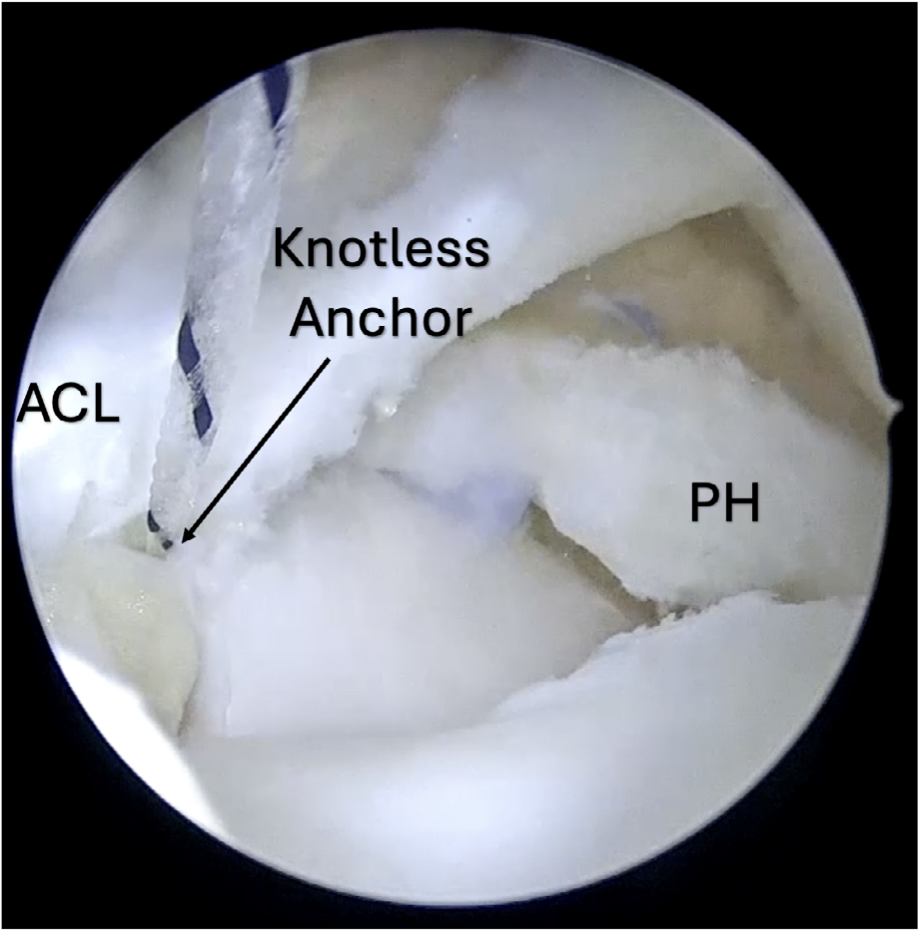
Left knee viewed from a supine, standard anterolateral portal showing the knotless anchor placed through the anteromedial portal at the posterior root footprint, with the PH and ACL labeled for orientation. (ACL, anterior cruciate ligament; PH, posterior horn.)

**FIGURE 4:**
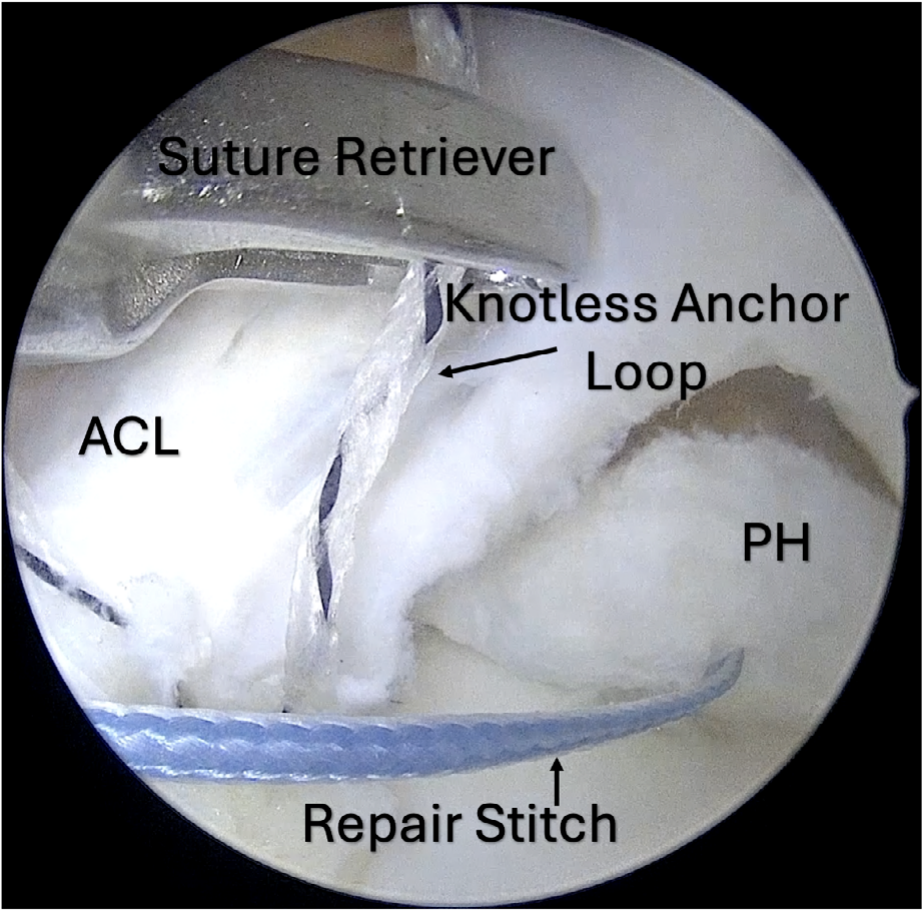
Left knee viewed from a supine, standard anterolateral portal showing the lateral meniscus PH being secured to the posterior root footprint using a 1.8‐mm knotless FiberTak anchor which entered through the high anteromedial portal, with the ACL and PH labeled for orientation. The anchor is passed antegrade into the posterior root footprint while avoiding a transphyseal trajectory or fixation across the physis. The repair suture is passed through the PH in a horizontal mattress configuration and secured within the knotless anchor, thereby recreating the native root attachment. (ACL, anterior cruciate ligament; PH, posterior horn.)

### Peripheral Stabilization

Three FiberStitch all‐inside repair devices (Arthrex) are placed above and below the meniscus posterior to the popliteal hiatus to establish posterior capsular attachments (Figure 5).

**FIGURE 5:**
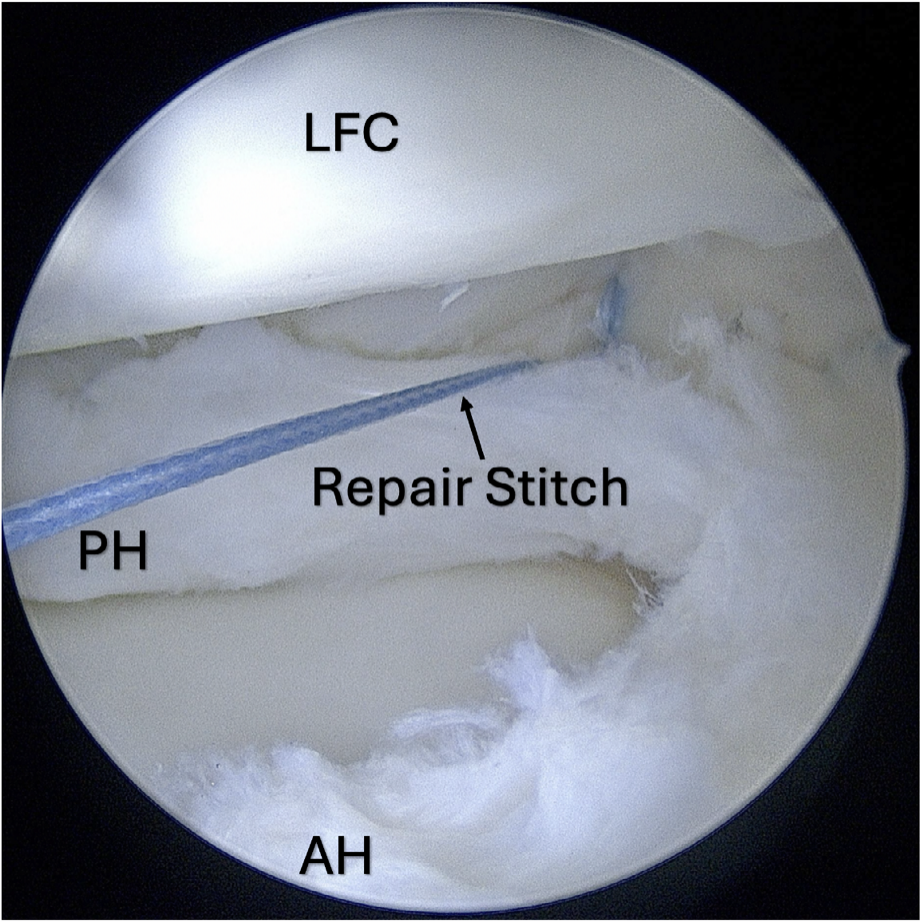
Left knee viewed from a supine, standard anterolateral portal showing the lateral meniscus being stabilized with the first of 3 all‐inside devices to establish posterior capsular attachments, with the AH, PH, and LFC labeled for orientation. (AH, anterior horn; LFC, lateral femoral condyle; PH, posterior horn.)

### Anterior Horn Stabilization

Using zone‐specific cannulas, two 2.0 FiberWire sutures (Arthrex) are passed from the anterior horn/body junction and tied over the anterior capsule to stabilize the anterior horn and complete circumferential stabilization (Figure 6).

**FIGURE 6:**
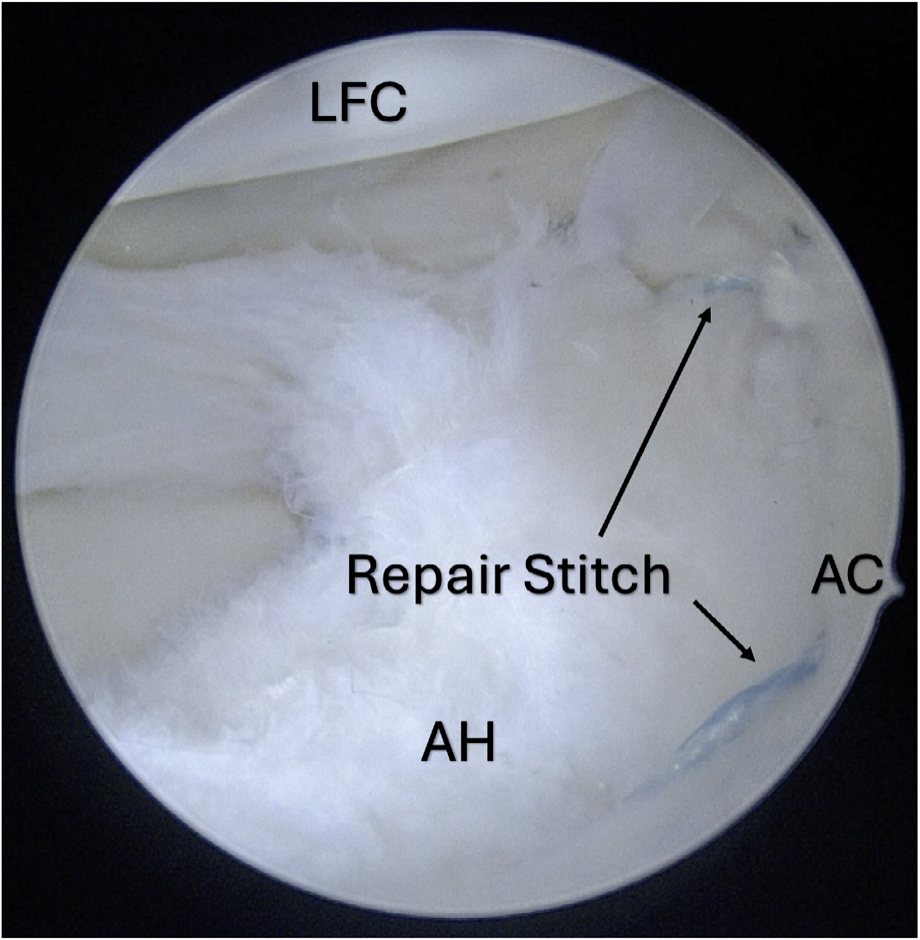
Left knee viewed from a supine, standard anteromedial portal showing 2 inside‐out vertical mattress repair sutures, which are placed using zone‐specific cannulas into the AH and body junction and tied over the AC to stabilize the AH, completing circumferential stabilization. The AH, AC, and LFC are labeled for orientation. (AC, anterior capsule; AH, anterior horn; LFC, lateral femoral condyle.)

### Biologic Augmentation

A PowerPick microfracture device (Arthrex) is used in the intercondylar notch to promote healing via marrow stimulation. On completion of all repairs, arthroscopy confirms a stable, well‐contoured lateral meniscus with normal mobility. The knee is thoroughly irrigated and drained.

### Postoperative Care

The operated knee is placed in a knee extension brace and remains non‐weight‐bearing for 4 weeks with no flexion past 90°. Physical therapy is initiated at 2 weeks postoperation focusing on quadriceps activation, with an additional 4 weeks of non‐weight‐bearing with no flexion past 90°. Full participation in sports, including running, is permitted at 6 months after the operation.

## DISCUSSION

The standard approach for DLM repair includes saucerization and repair using a combination of suture techniques that is determined by the location of the tear. However, the Wrisberg variant presents unique biomechanical challenges because of the absence of a native posterior root and capsular attachments, often resulting in global instability. In cases of complete posterior root detachment, reconstruction of the root is necessary to restore hoop stress transmission and preserve meniscus function.

Standard posterior root repair is most commonly performed using a transtibial pullout technique with favorable clinical outcomes.^7^ However, in skeletally immature patients, this approach may violate the proximal tibial physis. Although the true risk of clinically significant growth disturbance remains incompletely defined, iatrogenic physeal injury remains a concern in pediatric populations.

This technique offers several advantages, especially in the pediatric population. First, the use of a suture anchor eliminates the need to disrupt the tibial physis. Although traditional posterior root repairs have been done using the transtibial pullout method or suture anchor methods, a suture anchor technique in a pediatric Watanabe 3 DLM has not yet been published.^1^ A transtibial method requires drilling across the tibial physis, which poses a risk of growth disturbances in pediatric patients. This is something to be considered because the tibial physis contributes approximately 57% of tibial growth and 30% of total lower extremity growth.^8^, ^9^ Our technique offers a solution that maintains the integrity of the growth plate and repairs the posterior root attachment. Second, the use of an anchor‐first method allowed for real‐time tensioning of the root repair, which is not feasible with the standard transtibial method which is tensioned after passage of the suture. This allows for intraoperative retensioning, which may help optimize initial meniscal root positioning and repair stability, especially in the pediatric population. Suture anchor fixation in other soft‐tissue repairs, such as patellar tendon repair, has been shown to significantly reduce gap formation and load to failure, supporting the biomechanical rationale for this approach.^10^, ^11^ Third, the suture anchor method eliminates the risk of tunnel convergence as seen in the transtibial pullout method, making the repair procedure safer and simpler in the pediatric knee.^12^ Additionally, the knotless suture avoids the sliding motion inherent to the transtibial tunnel technique. The sliding motion decreases cell viability at the repair site which can compromise fixation. The knotless anchor thus provides a more stable form of fixation that may help preserve the biological environment and initial repair integrity in a pediatric patient.

Recent series have shown favorable outcomes after saucerization combined with meniscal repair and stabilization procedures in DLM surgery.^13^, ^14^, ^15^ Contemporary literature continues to emphasize the preservation of native meniscal biomechanics whenever possible.

This technique has several limitations. Anchor placement in the posterior root footprint can be technically difficult, especially in the smaller working space of the pediatric knee. To facilitate the proper angle of approach for the posterior root repair, a high anteromedial portal was created. This positioning of this portal allows for appropriate trajectory and therefore proper anchor insertion. Accurate portal placement, specifically the creation of a high anteromedial portal, is critical to achieve safe trajectory and avoid iatrogenic cartilage or neurovascular injury.

Additionally, there is a lack of long‐term clinical outcome data evaluating physeal‐sparing anchor‐based posterior root reconstruction in Wrisberg variant DLM, particularly in immature patients. Further longitudinal clinical studies are required. Finally, cost considerations may exist with the use of specialized knotless anchor implants compared with traditional suture‐based transtibial techniques.

Preservation of the tibial physis is paramount for pediatric patients obtaining DLM and posterior root repairs. Although there is limited data on cases in which this is encountered, the technique provides a growth plate‐conscious alternative for the management of this rare meniscal variant. This technique employs a knotless retensionable suture anchor that allows for repair of a Wrisberg variant DLM without disrupting the tibial physis and allows for intraoperative retensioning. Although technically demanding and requiring further long‐term validation, this technique provides a growth plate‐conscious alternative for the management of a rare and unstable meniscal variant. The pearls and pitfalls are shown in Table 1. The advantages and disadvantages are shown in Table 2.

**TABLE 1:** Pearls and Pitfalls of Physeal‐Sparing Knotless Anchor Reconstruction for Wrisberg Variant Discoid Lateral Meniscus

**Pearls**	**Pitfalls**
Confirm complete absence of posterior root and capsular attachments during diagnostic arthroscopy	Failure to fully assess instability may lead to undertreatment if only saucerization is performed
Create a high anteromedial portal to optimize trajectory to the posterior root	Improper portal placement may lead to poor anchor trajectory or cartilage injury
Thoroughly prepare and debride the posterior root to bone before anchor placement	Inadequate preparation may compromise fixation and healing
Use anchor‐first, retensionable fixation to optimize root tension intraoperatively	Overtensioning may alter meniscus biomechanics or restrict range of motion
Confirm stability and hoop tension arthroscopically prior to final fixation	Failure to confirm stability may lead to persistent instability

**TABLE 2:** Advantages and Disadvantages of Physeal‐Sparing Knotless Anchor Reconstruction for Wrisberg Variant Discoid Lateral Meniscus

**Advantages**	**Disadvantages**
Avoids transphyseal drilling and protects the tibial physis	Technically demanding, particularly in pediatric knees
Allows anatomic reconstruction at the native posterior root footprint	Limited long‐term clinical outcome data
Retensionable construct allows for intraoperative tension adjustment	Precise portal placement and anchor trajectory required
Eliminates risk of tibial tunnel convergence	Increased implant‐related cost
Provides stable knotless fixation construct	Less forgiving if the anchor placement is suboptimal

## DISCLOSURES

The authors (S.R., A.H., D.G., G.P., R.M.) declare that they have no known competing financial interests or personal relationships that could have appeared to influence the work reported in this article.
